# An Improved Roadside Parking Space Occupancy Detection Method Based on Magnetic Sensors and Wireless Signal Strength

**DOI:** 10.3390/s19102348

**Published:** 2019-05-21

**Authors:** Liangliang Lou, Jinyi Zhang, Yong Xiong, Yanliang Jin

**Affiliations:** 1 Key Laboratory of Specialty Fiber Optics and Optical Access Networks, Joint International Research Laboratory of Specialty Fiber Optics and Advanced Communication, Shanghai Institute for Advanced Communication and Data Science, School of Communication and Information Engineering, Shanghai University, Shanghai 200072, China; liangliang.lou@mail.sim.ac.cn (L.L.); zhangjinyi@shu.edu.cn (J.Z.); wuhaide@shu.edu.cn (Y.J.); 2 Shanghai Institute of Microsystem and Information Technology, Chinese Academy of Science, Shanghai 200050, China

**Keywords:** vehicle detection, magnetic sensor, received signal strength, sensor fusion

## Abstract

Smart Parking Management Systems (SPMSs) have become a research hotspot in recent years. Many researchers are focused on vehicle detection technology for SPMS which is based on magnetic sensors. Magnetism-based wireless vehicle detectors (WVDs) integrate low-power wireless communication technology, which improves the convenience of construction and maintenance. However, the magnetic signals are not only susceptible to the adjacent vehicles, but also affected by the magnetic signal dead zone of high-chassis vehicles, resulting in a decrease in vehicle detection accuracy. In order to improve the vehicle detection accuracy of the magnetism-based WVDs, the paper introduces an RF-based vehicle detection method based on the characteristics analysis of received signal strengths (RSSs) generated by the wireless transceivers. Since wireless transceivers consume more energy than magnetic sensors, the proposed RF-based method is only activated to extract the data characteristics of RSSs to further judge the states of vehicles when the data feature of magnetic signals is not sufficient to provide accurate judgment on parking space status. The proposed method was evaluated in an actual roadside parking lot and experimental results show that when the sampling rate of magnetic sensor is 1 Hz, the vehicle detection accuracy is up to 99.62%. Moreover, compared with machine-learning-based vehicle detection method, the experimental results show that our method has achieved a good compromise between detection accuracy and power consumption.

## 1. Introduction

In recent years, with the rapid increase in the number of private vehicles, the problem of parking has seriously affected citizens, and the continued construction of parking lots has been unable to solve the problem of parking difficulties. Since the number of parking spaces in the city cannot be increased without limit, the best way is to use technology to manage the existing parking spaces to improve the utilization rate of the existing parking spaces [1]. As a result, Smart Parking Management System based on Internet of Things (IoT) technology has been applied in many cities [2]. However, the realization of such systems requires a large amount of parking space status data to support the stable operation of the SPMS.

At present, there are many technologies to realize parking space status detection, but every technology has its drawbacks. Inductive-loop vehicle detectors [3] require a wired power supply which makes them difficult to install and maintain. Ultrasonic-based vehicle detector [4] and infrared-based vehicle detector [5] have the advantages of low cost, small size and high stability, which make them widely used in indoor SPMS. However, such vehicle detectors are susceptible to environmental factors such as temperature, light, rain, snow, etc., resulting in that they are rarely used in outdoor SPMS. The vision-based vehicle detectors [6] have been widely used in a variety of scenarios because they can provide users with rich and intuitive image information. However, the vision-based vehicle detectors are rarely used in roadside parking lots to achieve parking spaces status, because cameras are susceptible to rain, fog and lighting conditions, etc. Additionally, their current costs are relatively higher than other sensors. In recent years, the laser range finders (LIDAR) [7] and millimeter-wave radar [8] have been widely used in autonomous cars. Since the power consumption of these sensors is very high, their applications in battery-powered vehicle detectors are limited.

The magnetic sensor [9] has the advantage of high sensitivity, small size, flexible installation, strong anti-interference ability, and is basically not affected by environmental factors such as light, rain and snow, etc. The magnetic-sensor-based WVDs are widely used in roadside parking lots to get status information of the parking spaces. However, the magnetic signals are not only susceptible to the adjacent vehicles, but also affected by the magnetic signal dead zone of high-chassis vehicles. As a result, the vehicle detector based on magnetic sensor may make a wrong judgment or decision on the roadside parking space status under the above two circumstances.

In order to improve the vehicle detection accuracy of WVDs based on single magnetic sensor, machine-learning-based vehicle detection methods were proposed in [10,11,12,13,14,15]. This kind of vehicle detection algorithm mainly uses the machine learning method to analyze and identify the magnetic disturbance signal caused by vehicles to realize the detection of vehicle status. However, the shortcoming of the machine-learning-based vehicle detection methods is that they require the magnetic sensor to collect a large amount of magnetic data at a higher sampling rate and spend lots of time to process the collected magnetic data. As a result, the vehicle detector needs to spend most of its energy on magnetic signal acquisition and analysis. In addition, due to the limited resources of low-power microcontrollers, such algorithms are difficult to be implemented in the low-power microcontrollers.

Simultaneously, the multi-sensor data fusion vehicle detection methods combining magnetic sensor with optical sensor [16] and accelerometer sensor [17] were proposed to improve vehicle detection accuracy, and experimental results showed that those methods did improve vehicle detection accuracy. However, the performance of optical sensors is susceptible to environmental factors, such as dust, rain and fog, etc., which may cause the vehicle status to be misjudged under certain critical conditions. Since the parked vehicle cannot produce significant vibration signals on the parking space that can be sensed by accelerometer sensors and hence the vehicle detection method based on the magnetic sensor and accelerometer sensor is also not suitable for the roadside SPMS to realize the detection of parking space status. Moreover, adding new sensors to the vehicle detectors will increase their cost.

According to the theory of electromagnetic waves, when an obstacle appears in the wireless path, the intensity of the electromagnetic waves signals is attenuated, resulting in the radio waves energy received by the receiver is much smaller than that when there is no obstacle. The RF-based vehicle detection method was proposed in [18,19], where the authors deployed Wi-Fi access points on a roadside and used laptops to receive the Wi-Fi signals transmitted by the access points on the other roadside. As a result, the algorithms of vehicle detection and speed estimation are realized by analyzing the changes in Wi-Fi received signal strengths which are disturbed by the vehicles running on the road. This provides a new way for vehicle detection methods, however this method cannot achieve accurate detection when multiple vehicles exist in a small area.

In order to reduce the vehicle detection error rate when the magnetic signals are interfered by adjacent vehicles or the dead zones of high-chassis vehicles an improved vehicle detection method based on the combination of magnetic signals and received signal strengths is proposed in the paper. The principle of the proposed method is that when the data feature of the magnetic signals is not sufficient for WVDs to make an accurate decision on the status of vehicle, the data characteristics of the received signal strengths generated by the wireless transceivers in the WVDs and wireless access points (WAPs) will be used to make a further judgment on the vehicle status, thereby improving the vehicle detection accuracy of WVDs used in the roadside parking lots. Therefore, the proposed method does not add any cost to the wireless vehicle detector.

In addition, compared with the machine-learning-based vehicle detection methods mentioned above, the magnetic sensor in our proposed method is used to capture the magnetic disturbance signals caused by the vehicles without excessive extraction and analysis. As a result, the proposed method can run stably and reliably when the sampling rate of the magnetic sensor is only 1 Hz, resulting in a significant reduction in the overall power consumption of the wireless vehicle detectors, thereby effectively extend service life of the battery-powered WVDs. Therefore, the proposed method has very important practical significance.

The remaining of this paper is organized as follows: Section 2 analyses the characteristics of magnetic signals and received signal strengths. Section 3 proposes the vehicle detection method. The experimental verification is presented in Section 4. Finally, Section 5 contains the conclusion.

## 2. Characteristics of Magnetic Signals and Received Signal Strengths

### 2.1. System Overview

The wireless sensor network (WSN) is a group of specialized transducers with a communications infrastructure for monitoring and recording conditions at different locations and consists of multiple sensor nodes, each of which is small, lightweight, portable and battery-powered. Every sensor node is made up of a transducer, microcontroller, transceiver and power source. The transducer produces electrical signals based on perceived physical effects and phenomena. The microcontroller processes and stores the output of the sensor. The transceiver receives commands from a central computer and transmits data to that computer. The power for each sensor node comes from a battery [20,21].

**Figure 1:**
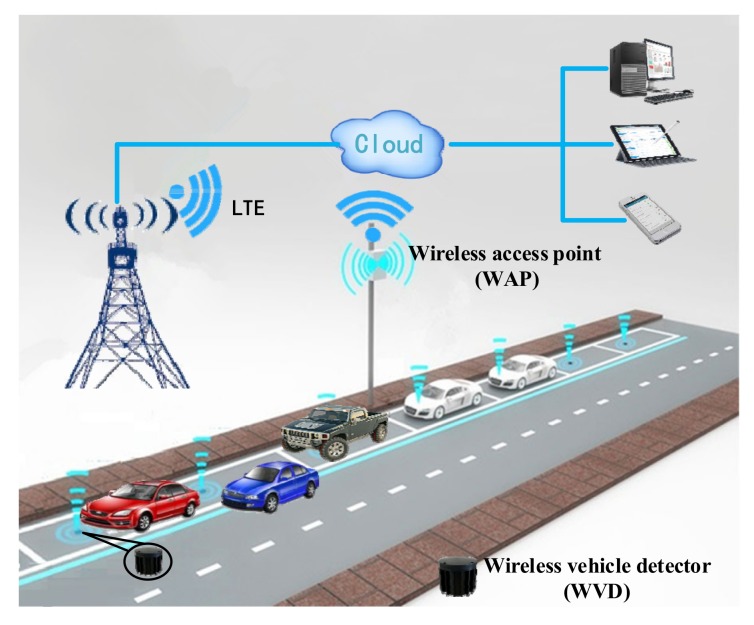
Roadside SPMS architecture.

In general, the roadside smart parking management system is a typical wireless sensor network. The roadside SPMS is composed of several wireless access points, multiple wireless vehicle detectors and a cloud server. The wireless vehicle detectors are installed in the middle of parking spaces to realize the accurate perception of the status of the vehicles on the parking spaces. The wireless access points, are installed within the effective communication distance of the wireless vehicle detectors. The wireless access points mainly undertake the establishment and maintenance of the wireless sensor network, receive and forward detection results from the vehicle detectors to the cloud server. Therefore, the status of parking spaces can be displayed on various terminals such as personal computers, smart phones and smart tablets, etc. The architecture of a roadside SPMS as shown in Figure 1.

### 2.2. Defects of WVD Based on Single Magnetic Sensor

**Figure 2:**
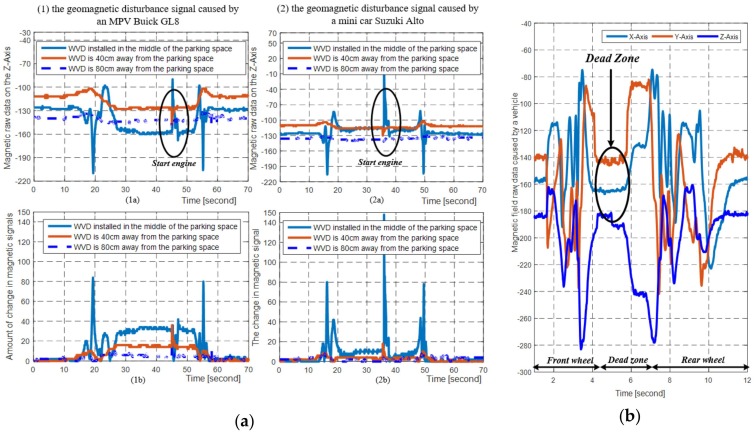
The raw magnetic data collected by the magnetic sensor 3D MagIC. (**a**) The magnetic disturbance data collected by the magnetic sensors at different distances during a vehicle parking process, and the sampling rates of magnetic sensors are 20 Hz; (**b**) The magnetic disturbance signals obtained by the magnetic sensor when an MPV Buick GL8 slowly passes over a magnetic sensor, and the magnetic sensor sampling rate is 100 Hz.

According to [22,23], we can achieve vehicle detection by analyzing the data characteristics of magnetic disturbance signals caused by the vehicles on the WVDs. However, the magnetic signals are easily affected by the adjacent vehicles, including the nonstandard parked vehicles on the adjacent parking spaces and irregularly traveling vehicles on adjacent lanes, such as the red and blue cars shown in Figure 1. In addition, the magnetic signal dead zone of high-chassis vehicles also affects the performance of the WVDs. In order to analyze the data characteristics of magnetic signals affected by different types of vehicles, we installed a WVD in the middle of a parking space. Moreover, we also installed a WVD at a distance of 40 cm and 80 cm from the parking space respectively. The collected raw magnetic data is shown in Figure 2.

From Figure 2a, we can see that the adjacent vehicles do have an influence on the magnetic signals, and different vehicle types have different influences on the magnetic signals. Therefore, for the threshold-based vehicle detection method, it is very difficult to select a suitable threshold that can be used in detecting all vehicle types.

Moreover, from Figure 2b, we can find that the magnetic disturbance signals affected by the engine and axles of vehicle are the strongest, while the magnetic disturbance signal is the weakest between the front and rear wheels. Hence, it is possible for the vehicle detector based on single magnetic sensor to make a wrong decision on the status of the vehicle when the sensing region of the vehicle detector is between the front and rear wheels. This problem is particularly prominent in the vehicles with high chassis, such as sport utility vehicles (SUVs), multi-purpose vehicles (MPVs) and pickups, etc.

### 2.3. Radio Signal Mathematical Model

According to the theory of electromagnetic wave propagation that when an electromagnetic wave encounters an obstacle, the electromagnetic wave can bypass the obstacle in the form of diffraction when physical size of the obstacle is similar to that of the electromagnetic wave. Similarly, if the physical size of obstacle is much larger than that of the wavelength of the electromagnetic wave, the electromagnetic wave will refract at the boundary of the obstacle or directly penetrate the obstacle. In any case, the energy of the electromagnetic waves will be affected due to the presence of obstacles such as vehicles.

In telecommunication, the free-space path loss (FSPL) is the attenuation of radio energy between the transmitted radio signal and the received radio signal which caused by an obstacle in free spaces. The FSPL formula derives from the Friis transmission formula [24]:

(1) Pr=PtDtDrλ4πd2,

where $P_{r}$ is the received power, $P_{t}$ is the transmit power, $D_{t}$ is the gain of the transmit antenna, $D_{r}$ is the gain of the receive antenna, d is the distance between the receiver and the transmitter, λ is the wavelength of electromagnetic wave. The FSPL formula can be used to describe a radio loss value between the transmit and receive antenna. In the roadside SPMS, the radio loss between the WVDs and WAPs comes from the influence of the vehicle present in the radio environment. Assuming that the directivity for the transmit and receive antennas are isotropic and therefore unity, a convenient way to express FSPL is in terms of dB [25]:

(2) $$FSPL\left(db\right)=10log_{10}\left({\left(\frac{4\pi d}{\lambda }\right)}^{2}\right)=20log_{10}\left(d\right)+20log_{10}\left(f\right)-147.55,$$

## 3. Implementation of Our Proposed Method

### 3.1. Hardware Setup of WVD

**Figure 3:**
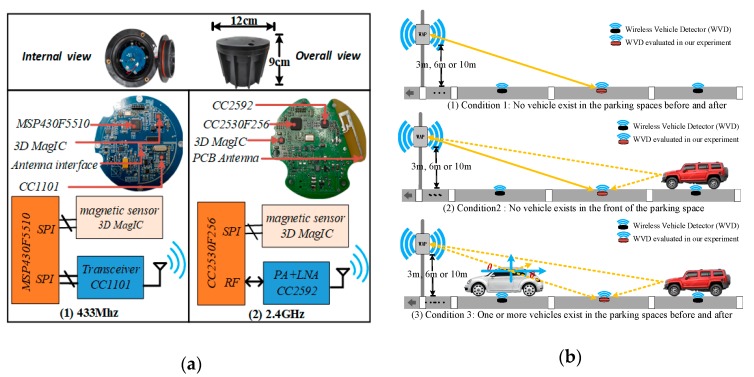
The hardware setups and different evaluation scenarios. (**a**) The hardware implementation of two types of wireless vehicle detector prototypes; (**b**) Three different evaluation and verification scenarios.

Generally, the typical wireless vehicle detector consists of a magnetic sensor, a wireless transceiver, a low-power microcontroller and a battery. According to Equations (1) and (2), we can find that the amount of attenuation depends on the radio frequency and distance. Therefore, we design two types of WVD prototypes with different radio frequencies to evaluate the performance of our proposed method, and the hardware setups shown in Figure 3a. The magnetic sensor used in our prototypes is the 3D MagIC produced by the PNI Company, Santa Rosa, CA, USA, the battery used in our prototypes is ER34615M with the nominal capacity of 15,000 mAh and the open voltage is 3.6 V.

The implementation block diagram of the WVD prototype with 433 MHz radio frequency is shown in Figure 3a1, and the low-power microcontroller MSP430F5510 is used to control the wireless transceiver chip CC1101 to achieve wireless communication and the maximum transmission power is 13 dBm. Similarly, the implementation block diagram of WVD prototype with 2.4 GHz radio frequency shown in Figure 3a2. From Figure 3a2, the wireless data transmission and reception are realized through the chip CC2530 which is a true system-on-chip (SoC) solution for IEEE 802.15.4. Since the maximum transmission power of CC2530 is only 4.5 dBm, we have added a range extender chip CC2592 to our evaluated circuit board to get better transmission performance, and the wireless transmission power is up to 20 dBm.

### 3.2. Characteristic Analysis of Magnetic Signals and Received Signal Strengths

The main aim of the proposed method is to fuse the wireless RSSs and magnetic signals to achieve accurate status of the vehicles parked on the roadside parking spaces. Therefore, before the implementation of the proposed vehicle detection method, we need to collect and analyze the data feature of RSSs and magnetic signals. Therefore, we installed above mentioned two types of WVD prototypes in the real roadside parking space to obtain RSSs and the raw magnetic data.

**Figure 4:**
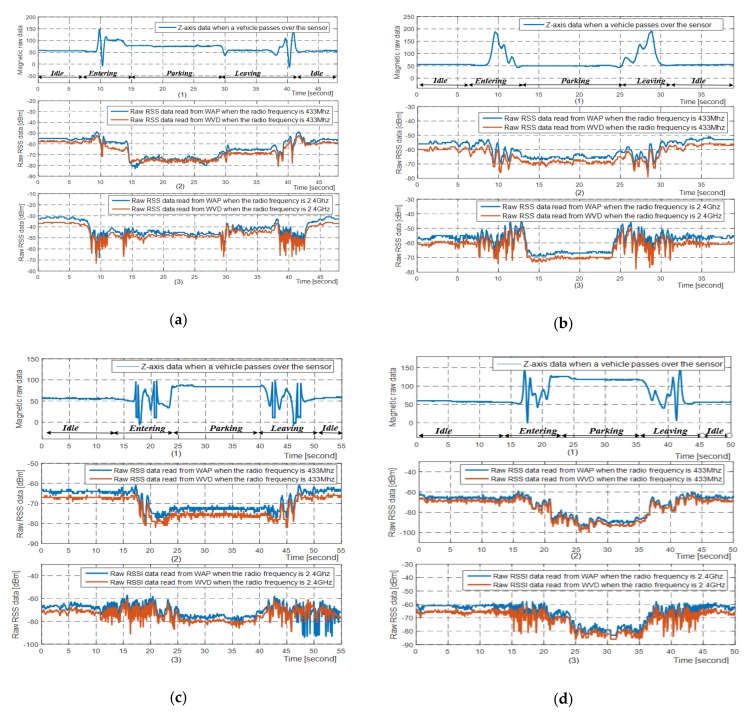
The raw magnetic raw data and raw RSSs affected by vehicle parking processes, and the tested vehicle is an MPV Buick GL8. (**a**) The WAP antenna is 3-meter-high and there are no vehicles in the parking spaces between WAP and WVD as the scenario shown in Figure 3b1; (**b**) The WAP antenna is 3-meter-high and there are some vehicles in the parking spaces between WAP and WVD as the scenario shown in Figure 3b3; (**c**) The WAP antenna is 6-meter-high and there are some vehicles in the parking spaces between WAP and WVD as the scenario shown in Figure 3b3; (**d**) The WAP antenna is 10-meter-high and there are some vehicles in the parking spaces between WAP and WVD as the scenario shown in Figure 3b3.

Generally, both the wireless transceivers in the WVD and WAP can generate an RSS when they receive a wireless data packet from the each other. In order to enhance the robustness of our proposed vehicle detection method, we need to collect the RSSs as much as possible. Hence, we append the RSSs generated by the WAP to the acknowledgement packets when the WAP receives messages from WVDs. In other words, when a complete wireless data interaction process is completed, the WVD can obtain two RSS data, one generated by itself and the other generated by the WAP. According to Equation (2), the electromagnetic wave energy received by wireless transceivers will be affected when vehicles appear in the wireless environment. Therefore, we collected and analyzed the data feature of RSSs and magnetic signals in three cases as shown in Figure 3b, which were: no vehicle on both front and behind, one or more vehicles on the behind, one or more vehicles on both front and behind. At the same time, considering that the antenna of the WAP at different heights may have some impacts on the RSSs, we installed the antenna of the WAP at different heights and carried out experiments respectively. The installation heights were: 3, 6 and 10 m as shown in Figure 3b too. The collected raw magnetic data and RSSs are shown in Figure 4.

From Figure 4 we can find that when a vehicle enters or leaves the sensing region of the vehicle detector, the magnetic signals and received signal strengths both change dramatically. Once the vehicle parking process is completed, the magnetic signals and the received signal strengths will remain relatively stable. From Figure 4b1 we can also find that the magnetic disturbance signals affected by the parked vehicle are so weak that may cause the WVDs with the single magnetism-based method to make a wrong decision on the status of the vehicle. Fortunately, from Figure 4b2,b3, the received signal strengths have changed significantly when the vehicle is present in the parking space, so it is completely feasible to use the data characteristics of the received signal strengths to further judge the state of the vehicle.

From Figure 4b–d we can find that the attenuation of the radio wave energy affected by the same vehicle increases as the installation height of the WAP antenna increases, and the possible cause of this is that the electromagnetic wave energy reflected by the vehicle body when the WAP antenna at height of 10 m is larger than that when the WAP antenna at height of 6 m and 3 m. We can also find that the higher the radio frequency, the more frequent changes occur when the vehicle enters or leaves the parking space.

From Figure 4, we can also find that the higher the frequency band of electromagnetic waves, the more frequent changes in the received signal strengths when vehicles enter or leave the parking space. However, the received signal strengths of the two frequency bands remain relatively stable after the vehicle is turned off. In summary, the data features of the RSSs generated by the wireless transceivers with the working frequency of 433 MHz and 2.4 GHz both can be used to detect the vehicle status.

### 3.3. Overview of Our Proposed Method

**Figure 5:**
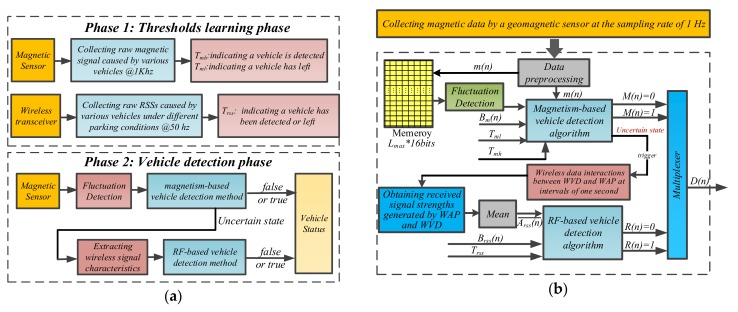
The implementation block diagram of proposed vehicle detection method. (**a**) Overview of our proposed vehicle detection method; (**b**) The implementation block diagram of our proposed fusion vehicle detection method.

According to the above analysis, if the amount of change in the magnetic signals is greater or less than the decision threshold, it can be considered that a vehicle has been detected or left, and this method will be called “magnetism-based method”. Similarly, if the amount of change in the received signal strengths is greater or less than a given threshold, a vehicle can be considered detected or left too, and we named this method “RF-based method”. We implemented our vehicle detection method in two phases, and the overview of our proposed vehicle detection method as shown in Figure 5a.

#### 3.3.1. Phase 1

From Figure 5a, three thresholds, $T_{mh}$, $T_{ml}$ and $T_{rss}$ are very important and will affect the accuracy of our proposed method. In order to get the three thresholds, we used magnetic sensors and wireless transceivers to collect the magnetic raw data and RSSs affected by different types of vehicles during their parking processes before our proposed vehicle detection method was implemented. Then, the three appropriate threshold values for our proposed method were derived from observation and analysis of large number of experimental results. The thresholds used in our proposed vehicle detection method are listed in Table 1.

#### 3.3.2. Phase 2

During this phase, the status of vehicles can be accurately detected by our proposed method. The magnetic sensor was periodically activated to collect the magnetic data and extract the data characteristics of magnetic signals to realize the vehicle detection. Since the magnetic signal dead zone exists between the front and rear wheels of the high-chassis vehicle, and magnetic signals are vulnerable to be affected by the irregularly parked vehicles on the adjacent parking spaces and slowly moving vehicles on the adjacent lane, the magnetism-based vehicle detection method may make wrong decisions on the status of parking spaces under above conditions. Therefore, in order to improve the detection accuracy, the RF-based vehicle detection method was activated to make a further judgment on the vehicle status when the magnetism-based method cannot obtain an accurate decision on them.

### 3.4. Implementation of Our Proposed Method

The vehicle detection method based on threshold algorithm has the advantages of low algorithm complexity, high reliability and low implementation difficulty. According to literature [15], if the appropriate thresholds and background references are selected, the threshold-based algorithm can detect the vehicle status accurately. Hence, the threshold-based algorithm is well suited for battery-powered WVDs to detect vehicle status and is widely used in many commercial wireless vehicle detectors based on the magnetic sensor, and our proposed vehicle detection method is also based on threshold-based algorithm.

In general, the drivers need to spend about 10–180 s to park vehicles into the roadside parking space and about 5–120 s to drive the vehicles out of the parking spaces. According to the Nyquist Theorem, if the sampling rate is above 0.2 Hz, the magnetic disturbance signals generated by the vehicle can be captured. Although the faster sampling rate can extract more rich magnetic signal characteristics information, it also means more energy is needed. Similarly, if the sampling rate is too low, the vehicle detector may not be able to capture the enough data feature of the magnetic signals, resulting in making a wrong decision on the status of the vehicle. In order to achieve a good trade-off between the power consumption and detection accuracy of wireless vehicle detector, the magnetic sensor sampling rate $SR_{m}$ used in our proposed method is 1 Hz.

According to Figure 5a2, we can divide the implementation of our proposed method into the following steps: Data preprocessing, Fluctuation Detection, Magnetism-Based Method Design, RF-Based Method Design and Two Methods Fusion Design.

#### 3.4.1. Data Preprocessing

The principle of the magnetism-based method is that only when the magnetic signals are large enough and last long enough, it will be determined as a vehicle arrival point. Similarly, the vehicle leave point is reached when the changes in magnetic signals are smaller than a threshold for a while [21]. Therefore, two windows, arrival detection sliding window and leaving detection sliding window, are used to check whether this criterions are met, and we have created a circular queue in the *RAM* of the low-power microcontroller to store the sampled magnetic data {$m\left(i\right),m\left(i+1\right),\ldots m\left(i+L_{max}-1\right)$} and the queue length:

(3) $$L_{max}=max\left(L_{e},L_{l},L_{v}\right)=max\left(W_{e}/SR_{m},W_{l}/SR_{m},W_{v}/SR_{m}\right),$$

where $L_{max}$ is the maximum length of the circular queue, $SR_{m}$ is the sampling rate of the magnetic sensor. $L_{e}$ and $L_{l}$ are the vehicle entering and leaving detection sliding window length respectively, $W_{e}$ and $W_{l}$ are the vehicle entering and leaving detection sliding window respectively and measure in units of second. $W_{v}$ is the fluctuation calculation window and $L_{v}$ is the length of data fluctuation calculation window, and the unit of $W_{v}$ is second too. The values of these parameters will be described in detail below.

#### 3.4.2. Fluctuation Detection

From Figure 4, we can see that the magnetic signals change drastically when a vehicle enters and leaves the parking space, and remain relatively stable when the vehicle is parked, so we can achieve the vehicle detection result by extracting the magnetic signal characteristics when the vehicle is parked in the parking space. Hence, we introduce a magnetic signal fluctuation detection mechanism in our proposed method, and the magnetism-based method is activated when the magnetic signal fluctuation is detected. We use the variance calculation algorithm to achieve the magnetic signals fluctuation detection result, and the data fluctuation calculation window $W_{v}$ is 10 s in our proposed method:

(4) $$F\left(k\right)=\left(\frac{\sum_{i=n-L_{v}+1}^{n}\left|m\left(i\right)-B_{m}\left(k\right)\right|}{L_{v}}>T_{ml}\right),$$

where k is the serial number of sampling point. $L_{v}$ is the length of data fluctuation calculation window. $B_{m}\left(n\right)$ is the current reference baseline of magnetism-based method. $T_{ml}$ is the vehicle leaving threshold of magnetism-based vehicle detection method. $F\left(k\right)$ is the binarized fluctuation detection result, the value changes from 0 to 1 indicating that a vehicle is entering or leaving the parking space. When $F\left(k\right)=1$, the value of $F\left(k\right)$ will be latched and cleared only when the status of the parking space changes. Equation (4) will only be executed until the circular queue has been filled.

#### 3.4.3. Magnetism-Based Method Design

**Figure 6:**
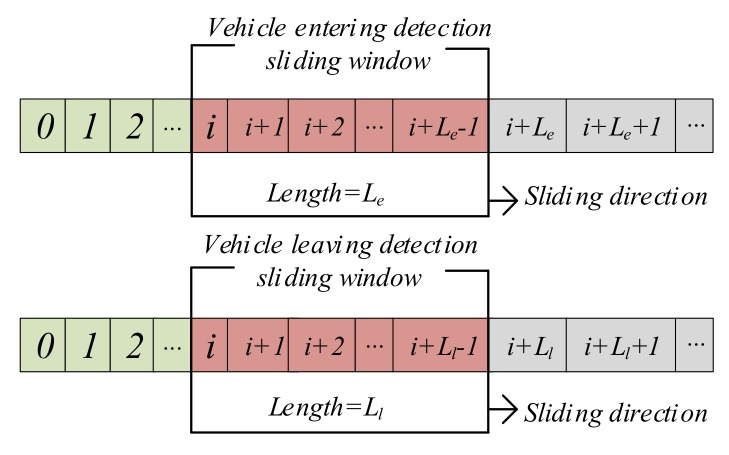
The implementation of sliding window algorithm for our method.

From Figure 2a, we can find that the magnetic signals are easily affected by adjacent moving vehicles, which may cause the vehicle state to be misjudged. In order to reduce the false detection probability caused by the interference from the adjacent moving vehicles, two detection windows shown in Figure 6 are introduced: vehicle entering detection sliding window $W_{e}$ and vehicle leaving detection sliding window $W_{l}$. Through the analysis of the general driver parking habits before, the vehicle entering detection sliding window $W_{e}$ size is 180 s and the vehicle leaving detection sliding window $W_{l}$ size is 120 s in our proposed method.

As described above, the magnetism-based method is activated to detect the vehicle status when the magnetic signals change drastically. Therefore, the value of the starting point i of the sliding window is equal to k in Equation (4). Here we define three signal states in vehicle detection process: 0, 1 and uncertain state. 0 means no vehicle exists on the parking space and 1 represents there is a vehicle on the parking space respectively, and the uncertain state indicates that the magnetism-based method cannot make an accurate decision on the vehicle status. The magnetism-based method can be expressed as:

(5) $$M\left(n\right)=\left\{\begin{array}{c} G\left(n\right),F\left(k\right)=1,\\ unchange,F\left(k\right)=0. \end{array}\right.$$

where $F\left(k\right)$ is magnetic signal fluctuation detection result, $M\left(n\right)$ is the vehicle detection result of the magnetism-based method, the value changes from 0 to 1 indicating that a vehicle has been parked on the parking space and vice versa. $G\left(n\right)$ can be obtained by the following formula:

(6) $$G\left(n\right)=\left\{\begin{array}{c} 1,\forall i\in \left(n-L_{e}+1,n-L_{e}+2,\ldots ,n\right)\left|m\left(i\right)-B_{m}\left(n\right)\right|>T_{mh},\\ 0,\forall i\in \left(n-L_{l}+1,n-L_{l}+2,\ldots ,n\right)\left|m\left(i\right)-B_{m}\left(n\right)\right|\leq T_{ml},\\ Uncertain\;state,\;otherwise. \end{array}\right.$$

where n is the serial number of sampling point. $m\left(i\right)$ is magnetic data value stored in the circular queue. $B_{m}\left(n\right)$ is the current reference baseline of magnetic signals. $T_{mh}$ and $T_{ml}$ are the vehicle entry and departure decision threshold respectively. Equation (6) will only be executed until the circular queue has been filled.

However, according to Equation (6), the magnetism-based method will enter an uncertain state when the amount of change in magnetic signals is between $T_{mh}$ and $T_{ml}$, which may be caused by the magnetic signal dead zone problem or the interference from adjacent vehicles. Therefore, we introduce an RF-based vehicle detection method for further detection to solve the afore mentioned uncertainty problem caused by the magnetism-based vehicle detection method. Similarly, the RF-based vehicle detection method is also based on the threshold comparison principle.

#### 3.4.4. RF-Based Method Design

Since the received signal strength data may be affected by environmental conditions, in order to eliminate the noise interference and improve the data reliability, we will continuously perform three wireless data interactions at intervals of one second to obtain six sets of wireless signal strength data when the RF-based method is activated. The RF-based method can expressed as follows:

(7) $$R\left(n\right)=\left\{\begin{array}{c} 1,\left|\bar{A_{rss}}\left(n\right)-B_{rss}\left(n\right)\right|>T_{rss},\\ 0,\left|\bar{A_{rss}}\left(n\right)-B_{rss}\left(n\right)\right|\leq T_{rss}. \end{array}\right.$$

where $\bar{A_{rss}}\left(n\right)$ is the average value of the six sets of wireless signal strength data which generated by the WVD and WAP during three wireless data interactions. $T_{rss}$ is the decision threshold of RF-based vehicle detection method. $R\left(n\right)$ is the vehicle detection result, the value changes from 0 to 1 indicating that there is a vehicle in the parking space and vice versa.

#### 3.4.5. Two Methods Fusion Design

According to the data sheets of wireless transceivers, under the condition of 3.6 V supply voltage of our evaluated circuit board, we can find that the transmit current can reach 150 mA when using CC2530 and CC2592, while the transmit current is about 30 mA when the CC1101 is used. Hence, if the transceiver module is activated all the time, the battery energy will be quickly depleted. In order to maximize the service lives of the WVDs, the proposed method utilizes the low power consumption magnetic sensor with 0.1 mA working current to trigger RF-based method to work under certain conditions to realize the detection of roadside parking space status. Through the above analysis, our proposed vehicle detection method for roadside SPMS based on the fusion of received signal strengths and magnetic signals can be expressed by following formula:

(8) $$D\left(n\right)=\left\{\begin{array}{c} M\left(n\right),\left(F\left(n\right)\neq Uncertain\;state\right),\\ R\left(n\right),otherwises. \end{array}\right.$$

where $D\left(n\right)$ is the vehicle detection result of our proposed method. According to Equation (8), the RF-based vehicle detection method is only activated when the magnetism-based vehicle detection method enters an uncertain state, otherwise the wireless transceiver module remains in sleep mode. According to the above analysis, the implementation block diagram of the proposed fusion vehicle detection method as shown in Figure 5b.

### 3.5. Adaptive Adjustment Algorithm for Reference Baselines

From Figure 5b we can see that the appropriate thresholds and reference values are the key parameters that determine the performance of our proposed method. The selection of three thresholds we have discussed above, this section will discuss the calculation algorithm for the reference values.

#### 3.5.1. Magnetism-Based Method Reference Background Calculation

Since the magnetic signals are easily affected by environmental factors such as temperature, which cause the reference background to drift and affect the vehicle detection accuracy. In this paper, we use the following weighted average method to update the reference background value:

(9) $$B_{m}\left(n\right)=\left\{\begin{array}{c} B_{m}\left(n-1\right)*\left(1-\alpha \right)+\alpha *m\left(n\right),D\left(n\right)=0\cap F\left(n\right)=0,\\ B_{m}\left(n-1\right),otherwise. \end{array}\right.$$

where n is the serial number of sampling point. Where α is a weighting coefficient and the value is 0.05 in our proposed method. $B_{m}\left(n\right)$ and $B_{m}\left(n-1\right)$ are the current and previous reference baseline of magnetic signals respectively. $m\left(n\right)$ is the current collected magnetic data on *Z*-axis. According to Equation (9), the reference background only be updated when no vehicle exists in the parking space and the magnetic signal is stable. Otherwise, it will remain unchanged.

#### 3.5.2. RF-Based Method Reference Background Calculation

The RF-based method, as same as the magnetism-based method, requires an adaptively adjusted background reference. In the roadside SPMS, the WVDs and WAPs will exchange heartbeat packets periodically for maintaining the network infrastructure after the accomplishment of wireless sensor network deployment. As a result, the RSSs can be generated by the WVDs and WAPs during the periodic heartbeat packet data interaction, which can be used to calculate the background reference of RF-based method. We also use the weighted average method to update the RF-based method reference background value:

(10) $$B_{rss}\left(n\right)=\left\{\begin{array}{c} B_{rss}\left(n-1\right)*\left(1-\alpha \right)+\alpha *\bar{A_{rss}}\left(n\right),D\left(n\right)=0\cap F\left(n\right)=0\\ B_{rss}\left(n-1\right),otherwise. \end{array}\right.$$

Where n is the serial number of sampling point. Where α is a weighting coefficient and the value is 0.05 in our proposed method. $B_{r\mathrm{ss}}\left(n\right)$ and $B_{r\mathrm{ss}}\left(n-1\right)$ are the current and previous reference baseline of RF-based method respectively. $\bar{A_{rss}}\left(n\right)$ is the average of the received signal strength generated by the WVD and the WAP during the periodic heartbeat packet data interactions.

## 4. Results

In order to evaluate the performance of our proposed vehicle detection method, we designed several vehicle detector prototypes with the method proposed in the paper and installed them in an actual roadside parking lot. The vehicle detector prototypes were installed in the center of the parking spaces. The experiments were conducted on the Pingcheng Road, Jiading, Shanghai, China.

### 4.1. Experimental Results

Through above analysis, due to the amount of change in the magnetic signals caused by the dead zone of vehicles is basically same as that affected by the non-standard parking vehicle on the adjacent parking space, which may cause the magnetism-based method to make a wrong judgement on the status of the parking space. In addition, the magnetic signal dead zone problem is particularly obvious in the high-chassis vehicles, such as SUVs, MPVs and pickups, etc. Therefore, we chose three types of vehicles to evaluate the reliability of the proposed method. The three types of vehicles were: a Volkswagen Santana 2000 sedan, a Suzuki Alto mini car, and a Honda CR-V sports utility vehicle.

From Figure 4b, we can find that when one or more vehicles exist between the evaluated WVD and the WAP, the higher the WAP antenna, the greater the radio energy attenuation. Considering the feasibility of construction, the installation height of the WAP antenna is 6 m in our experiment. In order to evaluate algorithm performance at different distances, two WVD prototypes were installed in the actual parking spaces. One was installed in the parking space which is about 100 m away from the WAP, and the other was installed in the parking space where is about 50 m away from the WAP.

We all know that different drivers have different driving habits. In order to evaluate the robustness of our proposed method, we chose three drivers with different driving ages to participate in our experiments. Moreover, we conducted the experiments in three cases as shown in Figure 3b, which were: no vehicle on both front and behind, one or more vehicles on the behind, one or more vehicles on both left and right. And the activation numbers of the RF-based detection method were record in our experiments to evaluate the energy consumption of the proposed method, the results are listed in Table 2.

From Table 2 we can find that the vehicle detection accuracy of our proposed method has exceeded 99.62% when the magnetic sensor sampling rate is 1 Hz. In general, the higher detection accuracy not only reduces the frequency of wireless transmission, but also improves the management efficiency of roadside parking lots.

From Table 2, we can also find that the activation probability of RF-based vehicle detection method in the vehicle detection accuracy evaluation process is about 9.2%. As described above, the magnetism-based vehicle detection algorithm will enter an uncertain state when the RF-based vehicle detection method is activated. The uncertain state may be caused by the interference from adjacent vehicles, or it may be caused by that the sensing range of the vehicle detector is just in the dead zone of the vehicle which has been parked on the current parking space. Therefore, it is very difficult for the magnetism-based vehicle detection method to make an accurate decision on the current parking space status under this situation. In other words, if the WVD equipped with the single magnetism-based vehicle detection method, the guaranteed detection accuracy of the WVD used in the roadside SPMS is only 90.8%. Therefore, compared with the single magnetism-based vehicle detection method, our proposed fusion vehicle detection method improves the detection accuracy by about 9.2%. So that the proposed vehicle detection method is very applicable to the roadside SPMS application.

### 4.2. Power Consumption Analysis

For battery-powered WVDs, lower average power consumption means longer service time, as a result, the evaluation of the power consumption characteristics has a guiding significance for battery selection. To calculate the average energy consumption of the proposed method, we need to know the average working current and duration of each activity performed:

(11) $$E=I*V_{DD}*t,$$

where E is the total energy consumption and the unit is mWh. I is the average working current and measure in unit of mA. $V_{DD}$ is the power supply voltage and the value is 3.6 V in our circuit board. t is duration of each activity performed and the units are hours:

(12) $$\left\{\begin{array}{c} E_{day}=E_{sleep}+E_{sensor}+E_{rf}+E_{mcu\;active},\\ E_{sensor}=I_{sensor}*V_{DD}*t_{sensor}*24*3600*SR_{m},\\ I_{rf\;avr}=I_{rf\;trans}*t_{rf\;trans}+I_{rf\;recv}*t_{rf\;recv},\\ E_{rf}=I_{rf\;avr}*V_{DD}*\left((1+T_{rss}*p_{rss}\right)*v_{num}+HB_{num}). \end{array}\right.$$

where $E_{day}$ and $E_{sleep}$ are the total energy consumption and standby energy consumption of a day respectively. $E_{sensor}$, $E_{rf}$ and $E_{mcu\;active}$ are the working energy consumption of the magnetic sensor, wireless transceiver and microcontroller respectively. $v_{num}$ is the total number of vehicles detected during a day. The other main parameters’ definitions and values are listed in Table 3. Because the microcontroller spends most of its time in sleep mode, only the magnetic sensor reading and writing operations, wireless data transmission and reception operations will wake up the microcontroller. Hence, we measured $E_{mcu\;active}$ together with the above several operations.

In order to estimate the average power consumption of each activity, we connected a 10 ohm resistor between a battery with the voltage of 3.6 V and the current board of vehicle detector prototypes, and used an oscilloscope to measure the voltage across the resistor and the duration of each activity. Hence, we can calculate the average power consumption of each activity by Ohm’s law and integral principle based on the voltage and duration obtained by the oscilloscope. Because the standby power consumption of the microcontroller is relatively low, we used a six-and-a-half digital multimeter Keysight 34465A to measure the standby power consumption of the microcontroller.

**Figure 7:**
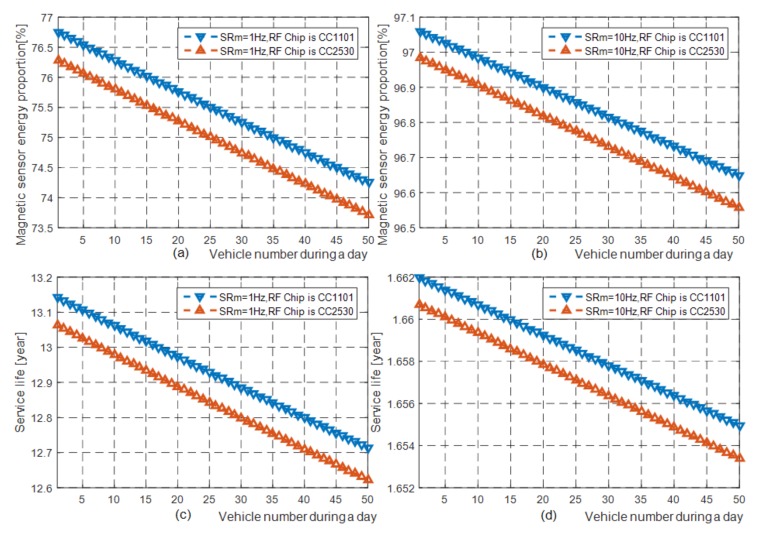
The relationship between the proportion of total energy consumed by the magnetic sensor and the number of vehicles detected per day, and the relationship between the WVD service years and the number of vehicles detected per day. (**a**) The proportion of the total energy consumed by the magnetic sensor in the two WVD prototypes with the radio frequency of 433 MHz and 2.4 GHz when the sampling rate of the magnetic sensor is 1 Hz. (**b**) The proportion of the total energy consumed by the magnetic sensor in the two WVD prototypes when the sampling rate of the magnetic sensor is 10 Hz. (**c**) The nominal serviceable years of the two prototypes when the sampling rate of the magnetic sensor is 1 Hz. (**d**) The nominal serviceable years of the two prototypes when the sampling rate of the magnetic sensor is 10 Hz.

The battery used in our vehicle detector prototype is ER34615M, which has an open circuit voltage of 3.6 V and a nominal capacity of 15,000 mAh. For the sake of simplicity, we assume that there is no retransmission in the wireless data interaction processes. With the above parameters, we can clearly get the total daily energy consumption of our proposed method and the proportion of the total daily energy consumption that consumed by the magnetic sensor as shown in Figure 7.

From Figure 7a,b, we can see that the energy consumption of the magnetic sensor accounts for a large proportion of the total energy consumption. In other words, the total energy consumption increases rapidly with the improvement of the magnetic sensor sampling rate, and vice versa. In summary, the above experiment results show that if the sampling rate of the magnetic sensor is reduced, the total energy consumption of the vehicle detector can be greatly reduced, so that the service life of vehicle detectors can be extended.

From Table 2, we can know that when the magnetic sensor sampling rate is 1 Hz, the vehicle detection accuracy is basically the same as the experimental results of the machine-learning-based vehicle detection method proposed in literature [12] (99.62% vs. 99.85%). However, the magnetic sensor sampling rate of the algorithm proposed in literature [12] is 10 Hz. From Figure 7b,d we can see that if the battery used in the WVDs in [12] is consistent with ours, their working life was no more than 2 years, while the working life of ours can exceed 10 years as shown in Figure 7c, therefore, our proposed vehicle detection method is very suitable for engineering application practice.

## 5. Conclusions

In the paper, the vehicle detection method based on the fusion of the magnetic signals and the received signal strengths is proposed. The proposed method does not require any modification on the hardware of wireless vehicle detectors and only requires some adjustments on the embedded software of wireless vehicle detectors, so the cost of the vehicle detector is not increased.

In addition, two types of wireless vehicle detector prototypes with different radio frequencies have been designed and implemented. Then, we have installed and evaluated the wireless vehicle detector prototypes with our proposed method in the real parking spaces. The experimental results show that the vehicle detection accuracy of our proposed method can exceed 99% when the magnetic sensor sampling rate is 1 Hz. Moreover, the energy consumption of our proposed method has been measured and estimated at last, compared with machine-learning-based vehicle detection methods, our method has achieved a good compromise between detection accuracy and power consumption, and it is very suitable for the battery-powered WVDs.

The lower sensor sampling rate means the lower energy consumption. However, we only evaluate the performance of the proposed method with 1 Hz magnetic sensor sampling rate in the paper. In order to extend the service life and improve the vehicle detection accuracy of WVDs as much as possible, in the future work, we need to build a mathematical model between power consumption, vehicle detection accuracy, sampling rate of magnetic sensor and wireless transceiver. As a result, we can choose an optimal combination of sensor sampling rates through the model according to parking habits of general drivers, and WVDs can achieve a good compromise between the power consumption and performance.

## Figures and Tables

**Table 1:** Three thresholds used in our proposed method.

Parameters	Description	Value
$T_{mh}$	vehicle entering decision threshold of magnetism-based detection method	20
$T_{ml}$	vehicle leaving decision threshold of magnetism-based detection method	5
$T_{r\mathrm{ss}}$	vehicle decision threshold of RF-based vehicle detection method	5

**Table 2:** Vehicle detection accuracy of our proposed method.

Case	Actual Number		Detected Number		RF-Based Method Activated Number		Detection Accuracy	
	50 m	100 m	50 m	100 m	50 m	100 m	50 m	100 m
no vehicle on both front and behind	199	175	199	174	15	18	100%	99.42%
some vehicles on the behind	202	195	201	195	21	18	99.50%	100%
some vehicles on both front and behind	188	169	187	168	17	15	99.46%	99.46%
overall	589	539	587	537	53	51	99.66%	99.62%

**Table 3:** Time duration and current consumption for each activity of the proposed method.

Parameters	Description	Value
$I_{mcu\;sleep}$	MCU standby current	1 μA
$I_{sensor\;sleep}$	Magnetic sensor standby current	1 μA
$I_{rf\;sleep}$	CC2530 or CC1101 standby current	1 μA
$I_{sensor\;}$	Magnetic sensor average operating current when the number of circuit oscillation cycles of the chip is 128	0.1 mA
$t_{sensor}$	Magnetic sensor operating time when the clock of sensor runs at nominally 32 MHz	1 ms
$p_{rss}$	Activation probability of RF-based method according to Table 2	9.2%
$T_{rss}$	Received signal strength sampling times by the RF-based method	3
$I_{rf\;trans}$	CC1101 or CC2530 average transmit current	30 or 154 mA
$t_{rf\;trans}$	CC1101 or CC2530 wireless data transmission time when data length is 32 bytes, and the data rates are both 250 kbps	2 ms
$I_{rf\;recv}$	CC1101 or CC2530 average operating current in receive mode	18 or 30 mA
$HB_{num}$	Number of heartbeat packets per day, and the heartbeat packet transmission period of wireless vehicle detector is 5 minutes.	288
$t_{rf\;recv}$	Wireless data reception window length	500 ms
